# Differential Expression of *DUB* Genes in Ovarian Cells Treated with Di-2-Ethylhexyl Phthalate

**DOI:** 10.3390/ijms21051755

**Published:** 2020-03-04

**Authors:** Da-Hye Lee, Jun-Hyeok Park, Jihye Choi, Kyung-Ju Lee, Bo-Seong Yun, Kwang-Hyun Baek

**Affiliations:** 1 Department of Biomedical Science, CHA University, Gyeonggi-Do 13488, Korea; po1290@hanmail.net (D.-H.L.); nymwns1@naver.com (J.-H.P.); gihea392@naver.com (J.C.); 2 Department of Obstetrics and Gynecology, Korea University Anam Hospital, Korea University College of Medicine, Seoul 02841, Korea; drlkj4094@gmail.com; 3 Department of Obstetrics and Gynecology, Gangnam CHA Hospital, College of Medicine, CHA University, Seoul 06135, Korea; bosungyun@chamc.co.kr

**Keywords:** DEHP, deubiquitinating enzyme, toxic environmental factor, multiplex RT-PCR, premature ovarian failure, qRT-PCR

## Abstract

Premature ovarian failure (POF) is defined as loss of ovarian function in women less than 40 years of age. The causes of POF are diverse and include environmental factors. Di-2-ethylhexyl phthalate (DEHP) is one factor that may cause POF. The ubiquitin-proteasome system maintains intracellular balance by promoting or inhibiting protein degradation. To investigate the differential expressions of *deubiquitinating enzyme* (*DUB*) genes in patients with POF, we developed two in vitro POF models by treating A2780 or OVCAR5 with DEHP. Using these models, a multiplex RT-PCR system for *DUB* genes was applied to identify biomarkers by comparing expression patterns and *DUB* mRNA levels; multiplex RT-PCR results were validated by qRT-PCR and Western blotting analyses. Observed differential expression levels of several *DUB* genes including *USP12*, *COPS5*, *ATXN3L*, *USP49*, and *USP34* in A2780 and OVCAR5 cells at the mRNA and protein levels suggest that they should be investigated as potential biomarkers of POF.

## 1. Introduction

Ubiquitination is an essential process that results in the degradation of dispensable proteins via the ubiquitin-proteasomal pathway (UPP) [1]. Protein ubiquitination is mediated by a series of enzymatic actions by ubiquitin-activating enzymes (E1), ubiquitin-conjugating enzymes (E2), and ubiquitin ligases (E3) [1,2]. As its name implies, deubiquitination is the inverse of ubiquitination, and deubiquitinating enzymes (DUBs) play a crucial role in protein stabilization by removing ubiquitin (Ub) from conjugated target proteins by hydrolyzing the isopeptide bonds of Ub-substrates. These ubiquitin-associated systems maintain intracellular protein balance by promoting or inhibiting degradation. The human genome codes for ~100 DUBs, most of which are classified as members of seven subfamilies; that is, ubiquitin C-terminal hydrolases (UCHs), ubiquitin-specific proteases (USPs), JAB1/MPN/Mov34 metalloenzymes (JAMMs), otubain proteases (OTUs), Machado-Joseph disease proteases (MJDs), permutated papain fold peptidases of dsDNA viruses and eukaryote monocyte (PPPDEs), and motif interacting with Ub-containing novel DUB family (MINDY) [3,4,5]. The biological functions of DUBs are not completely understood yet.

Premature ovarian failure (POF) involves loss of ovarian function in women less than 40 years old, which means ovaries do not produce normal amounts of estrogen and ovulation occurs sporadically and results in infertility [6]. POF can be present as primary amenorrhea with no menarche or as secondary amenorrhea with no menstrual period for 6 months or no menstrual period more than three times after the previous menstrual period [6]. The cause of POF is unclear, but it is known that both genetic and environmental factors are involved. Known causes include chromosomal abnormalities, autoimmune diseases, metabolic disorders, enzyme deficiencies, ovarian damage, and genetic diseases [7,8].

Environmental diseases are health disorders caused by physical, chemical, or biological environmental factors, often because of exposure to toxic environmental factors associated with lifestyle and occupational activities. Furthermore, it has been established that POF can be caused by toxic environmental factors [9].

Phthalates are one of the toxic environmental factors that may cause POF [10]. Phthalate esters are alkyl diesters of phthalic acid and are widely used as plasticizers, and are known to adversely affect two essential ovarian processes; that is, folliculogenesis and steroidogenesis [10]. Di-2-ethylhexyl phthalate (DEHP) is a member of the class of phthalates, and the relationship between DEHP and POF has been investigated, showing that the mechanistic link between the two has been elucidated [11,12,13]. One of the previous studies demonstrated that DEHP disrupts estrous cyclicity and PI3K signaling [14].

In order to investigate the differential expression of *DUB* genes and identify potential biomarkers of POF, we designed a multiplex PCR primer library of *DUB* genes [15] and treated two in vitro POF models, that is, A2780 and OVCAR5 ovarian cell lines, with DEHP, and confirmed results by qRT-PCR and Western blotting analyses [16]. Our results showed the differential expressions of mRNA and protein for several *DUB* genes including *Ataxin-3-like protein* (*ATXN3L*), *Ubiquitin specific peptidase 12* (*USP12*), *Ubiquitin specific peptidase 49* (*USP49*), *COP9 signalosome subunit 5* (*COPS5*), and *Ubiquitin specific peptidase 34* (*USP34*) in A2780 and OVCAR5 cells.

## 2. Results

### 2.1. Differential Expressions of DUBs by DEHP in Ovarian Cells

**Figure 1:**
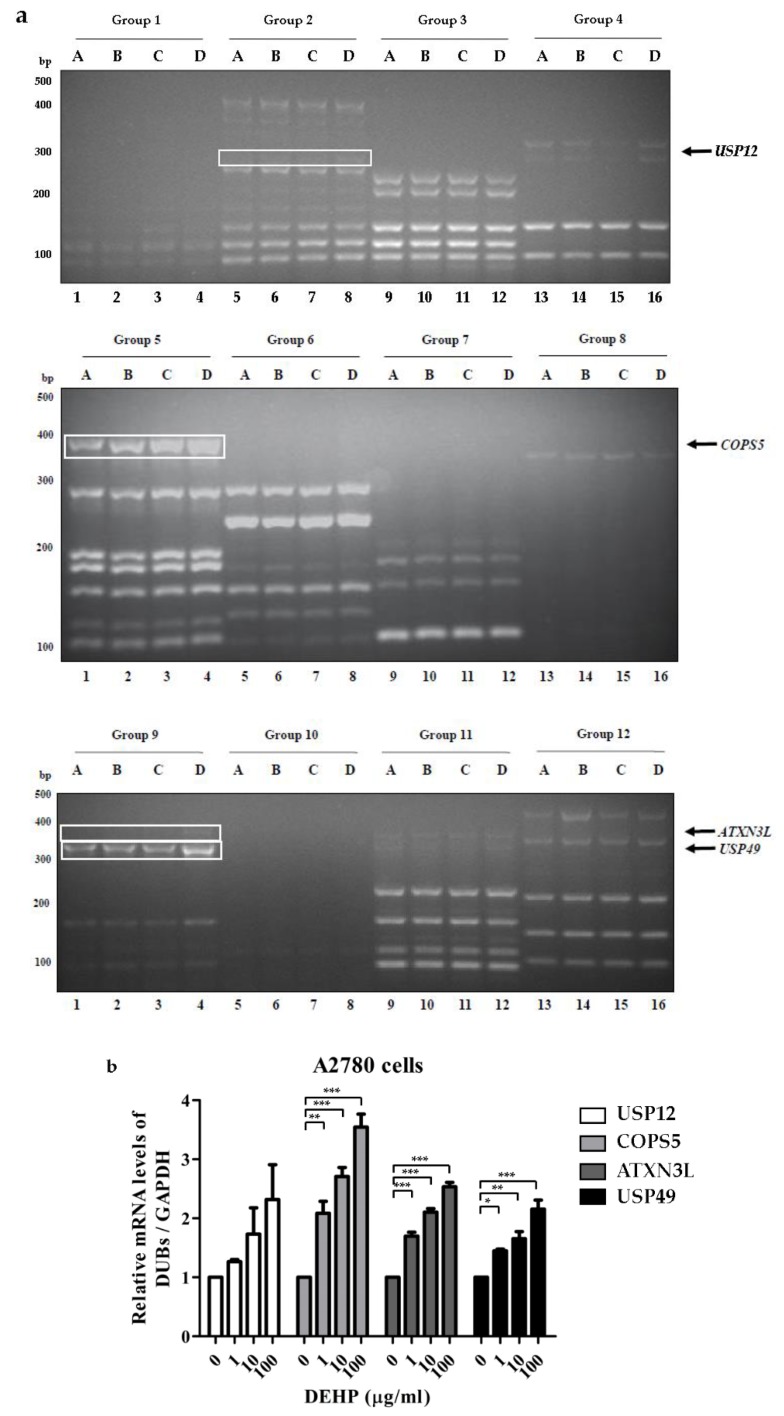
*Deubiquitinating enzyme* (*DUB)* gene screening in A2780 cells treated with DEHP using the multiplex RT-PCR primer library. (**a**) *DUB* genes were amplified by multiplex RT-PCR using 12 sets of primer library. (**b**) mRNA levels of *USP12*, *COPS5*, *ATXN3L*, and *USP49* were normalized with respect to *GAPDH*. The significances of differences were determined by one-way of variance. *p*-values are presented as * *p* < 0.05, ** *p* < 0.01, or *** *p* < 0.001.

**Figure 2:**
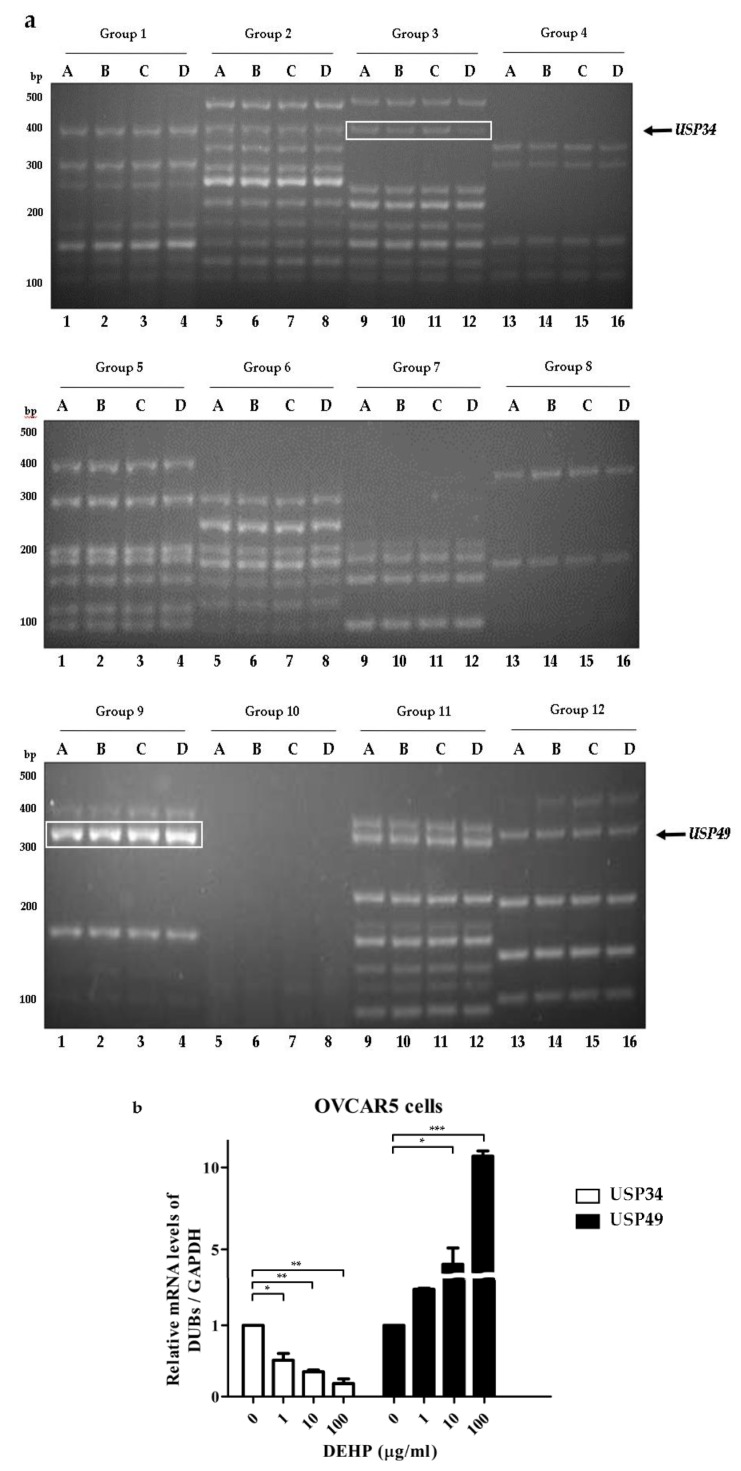
*DUB* gene screening in OVCAR5 cells treated with DEHP using the multiplex RT-PCR primer library. (**a**) *DUB* genes were amplified by multiplex RT-PCR using 12 sets of primer library. (**b**) mRNA levels of *USP34* and *USP49* were normalized with respect to *GAPDH*. The significances of differences were determined by one-way of variance. *p*-value are presented as * *p* < 0.05, ** *p* < 0.01, or *** *p* < 0.001.

To investigate *DUB* genes associated with DEHP, ovarian cell lines were treated with different concentrations of DEHP (Table 1) [16]. Multiplex RT-PCR was used to amplify DNA bands of *DUB* genes from cDNA synthesized from mRNA of A2780 and OVCAR5 cells treated with DEHP. When the multiplex RT-PCR was performed using all 12 sets of primers in the multiplex RT-PCR library, DNA amplification of *DUB* genes was detected (Figure 1 and Figure 2). Independent experiments were conducted at least three times. The results showed that the mRNA levels of *USP12*, *COPS5*, *ATXN3L*, and *USP49* were increased in A2780 cells (Figure 1 and Figure S1), and that the mRNA level of *USP49* was increased and that of *USP34* was decreased in OVCAR5 cells (Figure 2 and Figure S2) by DEHP exposure in a dose-dependent manner.

### 2.2. DEHP Exposure Influenced the mRNA Levels of Several DUB Genes in Ovarian Cells

**Figure 3:**
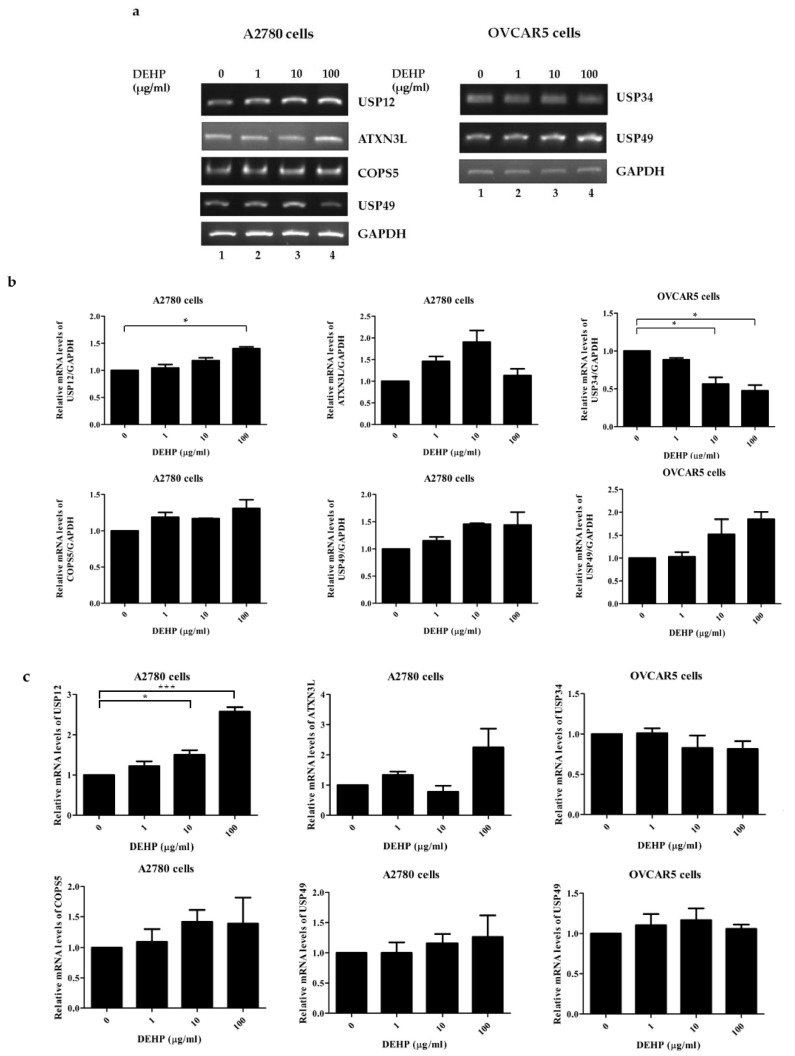
The mRNA expressions of *DUB* genes in DEHP-treated cells. (**a**) mRNA levels were assessed by RT-PCR in A2780 and OVCAR5 cells treated with different concentrations of DEHP. (**b**) The mRNA levels of *DUB* genes were normalized with respect to *GAPDH*. (**c**) The mRNA levels of *DUB* genes were examined by qRT-PCR in A2780 and OVCAR5 cells treated with different concentrations of DEHP. The significances of differences were determined by one-way of variance. *p*-values are presented as * *p* < 0.05, or *** *p* < 0.001.

To confirm the result of multiplex RT-PCR, RT-PCR and qRT-PCR were performed for selected *DUB* genes. The mRNA levels of *USP12*, *ATXN3L*, *COPS5*, and *USP49* were increased in A2780 cells by DEHP (Figure 3a and Figure S3a), and in OVCAR5 cells, the mRNA level of *USP34* was decreased and that of *USP49* was increased (Figure 3b and Figure S3b). Furthermore, these results were confirmed by qRT-PCR (Figure 3c and Table S1).

### 2.3. Up-Regulation of USP49 Protein by DEHP in Ovarian Cells

**Figure 4:**
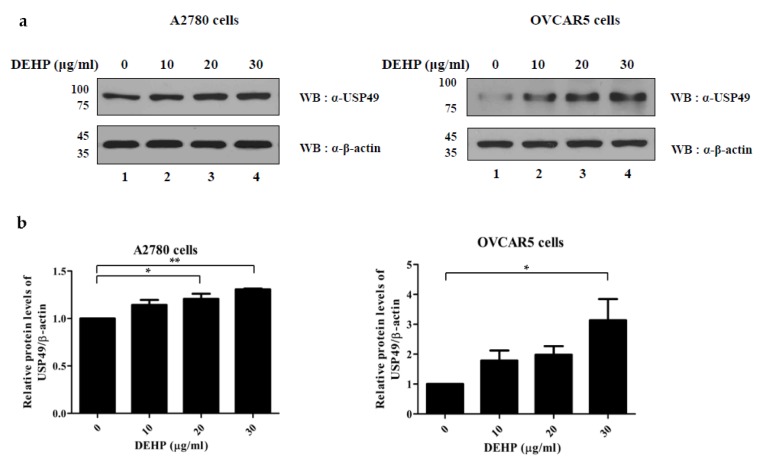
Expression of USP49 in the DEHP-treated cells. (**a**) Protein levels of USP49 in A2780 (left) and OVCAR5 (right) cells treated with DEHP were determined by Western blotting. (**b**) USP49 band intensities were normalized versus β-actin. The significances of differences were determined by one-way of variance. *p*-values are presented as *p < 0.05, ** *p* < 0.01.

As the mRNA level of *USP49* was found to be upregulated by DEHP in ovarian cells (Figure 3), we examined the effects of DEHP exposure on the protein level of USP49 in both cell lines by Western blotting analysis. As was expected, the protein levels of USP49 were upregulated in both cell lines by DEHP (Figure 4 and Figure S4).

## 3. Discussion

Exposure to toxic environmental factors is steadily increasing because they continue to be used in daily life, and as a result, the incidences of the diseases they cause are also increasing. On the other hand, progress is being made to develop products using less toxic materials. POF is a disease associated with toxic environmental factors in addition to genetic factors, and its incidence is also increasing. Therefore, we investigated mechanisms involving the ubiquitin-proteasome system initiated by exposure to DEHP in ovarian cell lines by using multiplex RT-PCR to screen for *DUB* genes differentially expressed after DEHP exposure.

ATXN3L is a Machado–Joseph disease protease (MJD), which contains the Josephin domain (JD), and JD containing proteins are known to have deubiquitination activity [17]. Furthermore, it has been shown that ATXN3L has greater deubiquitination activity than JD containing proteins [18]. Furthermore, ATXN3L is a DUB of Krüppel-like factor 5 (KLF5) and promotes breast cell proliferation, survival, and tumorigenesis [19]. In the present study, multiplex RT-PCR results showed that *ATXN3L* expression was increased in A2780 cells after DEHP exposure, suggesting that this would promote POF by promoting oocyte maturation, due to its cell proliferation capability [18].

USP12 is a ubiquitin-specific protease (USP) and a component of a complex containing USP1-associated factor 1 (UAF1) and WDR20. It also regulates androgen receptor (AR) in prostate cancer [20,21,22]. USP12 has been shown to regulate the ubiquitination level of histone H2A and H2B [23] and to deubiquitinate AR and MDM2, which regulate the p53-MDM2-AR-AKT signaling network. In addition, USP12 promotes cell survival, proliferation, tumorigenesis, and cell cycle progression by upregulating BMI-1, c-Myc, and cyclin D2 [24,25,26,27]. Our multiplex RT-PCR results showed that *USP12* expression was increased by DEHP in A2780 cells, and thus, the above-mentioned findings of its influence on cell cycle progression and proliferation suggest that it may induce POF by influencing oocyte maturation.

On the other hand, USP49 is also a ubiquitin-specific protease and regulates the initial pre-mRNA splicing by deubiquitinating histone H2B [28]. USP49 is also known to regulate tumorigenesis by regulating the involvement of p53 in DNA damage response and by controlling FK506-binding protein 51 (FKBP51) through the Akt signaling pathway [29,30]. Multiplex RT-PCR and Western blot analysis revealed that *USP49* was upregulated by DEHP in A2780 and OVCAR5 cells. These findings suggest that USP49 upregulation by DEHP induces cell death via p53 regulation, which subsequently leads to POF due to follicle depletion.

COPS5 is a JAB1/MPN/Mov34 metalloenzyme. Its expression increases during oocyte maturation and it is required for oocyte meiosis. COPS5 also regulates the maturation-promoting factor (MPF) activity [31]. Furthermore, progesterone receptor (PGR), which regulates ovulation, lies downstream of COPS5 [32,33]. COPS5 has been reported to control proliferation and inhibits the expression of p27 in serous ovarian cancer [34]. Our multiplex RT-PCR results showed that *COPS5* expression was increased in A2780 cells exposed to DEHP. Based on the above, COPS5 upregulation would be expected to promote POF by promoting oocyte maturation.

USP34 is a ubiquitin-specific protease that controls the stability of Axin and positively regulates Wnt/β-catenin signaling [35]. USP34 is required for DNA damage repair and regulates E3 ubiquitin-protein ligase RNF168, and recruits the DNA damage repair factors by ubiquitinating DNA double-strand breaks (DSBs) [36]. It has also been shown to negatively regulate T cell receptor (TCR) by inhibiting nuclear factor-κB (NK-κB) activation [37], and to be associated with polycystic ovary syndrome (PCOS), one of the most common endocrine disorders among women [38]. In the present study, multiplex RT-PCR results showed that *USP34* was downregulated by DEHP in OVCAR5 cells, which suggests a diminished ability to repair DNA damage and increase the risk of POF due to reductions in follicle numbers.

In summary, this study shows that DEHP changes the mRNA and protein levels of several *DUB* genes; that are, *USP12*, *ATXN3L*, *COPS5*, *USP49*, and *USP34* in the A2780 and OVCAR5 cells, and that it could potentially participate in the pathogenesis of POF. We suggest an in vivo study to be undertaken in a rodent model to further investigate the link between the DEHP-induced differential expressions of *DUB* genes and the pathogenesis of POF and to identify potential therapeutic targets.

## 4. Materials and Methods

### 4.1. Cell Culture

Human ovarian cancer cell lines, A2780 cells, were grown in the Roswell Park Memorial Institute-1640 medium (11875-093, RPMI-1640, Gibco BRL, Rockville, MD, USA) and OVCAR5 cells were grown in Dulbecco’s modified Eagle’s medium (11965084, DMEM, Gibco BRL, Rockville, MD, USA), containing 10% fetal bovine serum (26140079, FBS, Gibco, Grand Island, NY, USA), and 1% antibiotic-antimycotic reagent (15240062, Gibco, Tewksbury, MA, USA) at 37 °C in 5% CO2 atmosphere.

### 4.2. RNA Extraction and cDNA Synthesis

For RNA extraction, cells at 80–90% confluence in 60 mm dishes were lysed with TRIzol reagent (15596018, Ambion, Carlsbad, CA, USA). cDNA was prepared by reverse transcription with 1 μg of total RNA using the LaboPass cDNA Synthesis Kit (CMRTK001, Cosmogenetech Inc, Seoul, Korea) according to the manufacturer’s protocols.

### 4.3. Multiplex RT-PCR and qRT-PCR

For multiplex RT-PCR, we used 2× Multiplex PCR Smart Mix (SMP01-M25h, Solgent, Daejeon, Korea), 200 ng cDNA samples, and *GAPDH* as a control. PCR products were separated by 2% agarose gel electrophoresis and gels were stained with RedSafe DNA Stain (21141, Chembio, Medford, NY, USA) to visualize amplicons and confirmed the amplification of cDNA bands of expected sizes. Primers for the multiplex RT-PCR library (Table 2) were used to amplify the *DUB*-specific sequences (Ubiprotein Corp, Seongnam, Korea). mRNA expression levels were normalized versus *GAPDH* and analyzed using Image J v1.4.3.67 (National Institutes of Health, Bethesda, MD, USA). Expression levels of *DUB* genes in naïve A2780 and OVCAR5 cells were considered normal. Primers for qRT-PCR (Table 3) were used to compare the quantitative expression of *DUB* genes identified through multiplex RT-PCR (Ubiprotein Corp, Seongnam, Korea). qRT-PCR was performed using the StepOne Real-Time PCR System (Thermo Fisher Scientific, Inc., Waltham, MA, USA) according to the manufacturer’s instructions, and cDNA was amplified by reverse transcription using SYBR-Green PCR Master Mix (4309155, Applied Biosystems; Thermo Fisher Scientific, Inc., Waltham, MA, USA). Cycle threshold values of *DUB* genes were normalized to the endogenous reference gene *GAPDH*. The expression *DUB* genes levels were calculated using the 2^-∆∆CT^ method.

### 4.4. Western Blot Analysis

A2780 and OVCAR5 cells treated with DEHP were lysed using a lysis buffer (50 mM Tris-HCl (pH 7.5), 300 mM NaCl, 1 mM EDTA, 10% Glycerol, 1% Triton X-100). After resuspension of cells, samples were incubated for 20 min on ice and centrifuged at 13,000 rpm for 15 min. Then, cell lysates were mixed with 2X SDS loading buffer, boiled, and loaded into the SDS-PAGE gels. Separated proteins were transferred to polyvinylidene fluoride (PVDF) membranes (IPVH00010, Millipore, Billerica, MA, USA), which were incubated overnight with primary antibodies at 4 °C and then with secondary antibodies for 1 h at room temperature. Blots were visualized using the ECL reagent solution (LF-QC0101, Young In Frontier, Seoul, Korea).

### 4.5. Antibodies

Rabbit anti-USP49 polyclonal antibody (18066-1-AP) was purchased from Proteintech (Proteintech Group, Chicago, IL, USA) and mouse anti-β-actin monoclonal antibody (sc-47778) was purchased from Santa Cruz (Santa Cruz Biotechnology, CA, USA).

### 4.6. Statistical Analysis

Each experiment was performed at least three times. Densitometric analysis was performed for bands from all RT-PCR and Western blot results using a computer program Image J software (v1.4.3.67, National Institutes of Health, Bethesda, MD, USA) and the *t*-test using GraphPad Prism version 5 (GraphPad Software, La Jolla, CA, USA). ANOVA was performed by two-way analysis of variance. *p*-values are presented as * *p* < 0.05, ** *p* < 0.01, or *** *p* < 0.001 as indicated.

## Figures and Tables

**Table 1:** Concentration of di-2-ethylhexyl phthalate (DEHP) treatment.

Ovarian Cells	DEHP (di-2-ethylhexyl phthalate)
A	0 μg/mL (0 mM)
B	1 μg/mL (2.77 mM)
C	10 μg/mL (27.7 mM)
D	100 μg/mL (277 mM)

**Table 2:** Multiplex PCR primer set.

*DUB* Gene	Primer Sequence	Group	DUB Gene	Primer Sequence	Group
*Ataxin3*	F: GTC CAA CAG ATG CAT CGA CCA A	G4	*USP12*	F: GAA CTC TGA GTC TGG TTA CAT CCT	G2
	R: CGT CTA ACA TTC CTG AGC CAT C			R: GAG GAG CTG GTA TCT CTG ATT TCA	
*ATXN3L*	F: TCA GAA GAA AGT GAT GAG TCT GG	G9	*USP13*	F: ACC CAG CTG GAC AAT GGA GTC A	G2
	R: CTC TCA ATT GCT CTC GAA CTT G			R: CAG CTT GAT GTC ATT GTC CTG GA	
*BAP1*	F: TCC GTG ATC TGG GTC CTG TC	G11	*USP14*	F: TCA GTG TAT TCG TTC TGT GCC TGA	G2
	R: TCC CCG TCT TCT CTC TGC TG			R: CTC GCA TCA TTT GTA TCC AAC ATT CA	
*BRCC3*	F: GAG TTC AGA GTA TGA GAG AAT CG	G4	*USP15*	F: AAA CCT CGC TCC GGA AAG GGG A	G2
	R: CCT TTT CTT CTT GTT GTA ATT CCT G			R: CAG TTG GCA ACA GTA TGT AAT CCA A	
*COPS5*	F: GCA GTG GTG ATT GAT CCA ACA A	G5	*USP16*	F: AAA CTT TAG AAC CTG TGT GCA G	G3
	R: AGA CCT GAC CAG TGG TAT AGT C			R: CCT GAG AAT TTC TGC CAC AGC C	
*COPS6*	F: AGG TGT TCA AGG AGC TGG AGT T	G5	*USP17*	F: GAG CAA CGC AAG GAG AGC TCA AG	G8
	R: GGA AGA TCT GTG TGC TTG GTC A			R: AGG GTA CCT TCG ACT TTT CTG ACG	
*CYLD*	F: GCC AAG AAA AAG TCA CTT CAC CC	G11	*USP18*	F: ATT GGA CAG ACC TGC CTT A	G3
	R: TGC CTT TTT GCA GAA GGA ATC CT			R: AAG GAT TCC TTC ACC CGG ATC G	
*EIF3S3*	F: GTC CAA ACT CTT CAA ACC ACC A	G5	*USP19*	F: GTT CTT TCC TTC ATC GTC AGG GTC	G3
	R: AGT GAA CTC CTT GAT GTT CTG G			R: AGT GGG AGT AGC CAA GAG ATC ATG	
*EIF3S5*	F: TCT GCC TGG TCC TGC TCT TCC A	G4	*USP20*	F: TGG GCT CCT CTT CCA AGT TCT	G11
	R: TTG TCG ACA GTT CCC AAC AGG G			R: AGG TTT CAG GTC ATC GTC CTC T	
*JOSD1*	F: GTG AAT GTC ATT ATG GCA GCA C	G4	*USP21*	F: TGA CAA AGC CGG AAG TCC TGT A	G3
	R: TCC TCC AAC TCT GAT GAG CCT C			R: AAA GGG CTT CAC AGG TGC CAG A	
*JOSD2*	F: GTG TCT ACT ACA ACC TGG ACT C	G4	*USP22*	F: ACC AAC CAA ACG GGA GCT TG	G11
	R: ATG AAG TGC TGG CCT TTC CCA G			R: CCC AAG GTT GAT CAG CCC AC	
*MPND*	F: CGG GCA GAC CTT CAA CTC AC	G12	*USP24*	F: CCG ACA GTT GTC CGT GTC TG	G12
	R: CCC AGT GGT CTC CGA CTC TT			R: TCC GAA GCT GTA GGC ACG TA	
*OTUB1*	F: AGG AAC CTC AGC AGC AGA AGC A	G6	*USP26*	F: CAG CCA CCT GTG AGA CCT GGT AA	G8
	R: GTC TTG CGG ATG TAC GAG TAC T			R: CTG ATA ACT CTC CGC AAG TAA G	
*OTUB2*	F: CAT TCT TCG GGA CCA TCC TGA A	G6	*USP27*	F: CTC CAG CTT TAC GAT CGG TTT AAG	G1
	R: GTT CCC ATC CCC TTT GGT CTT			R: CCG AAA CAG CGA CGA CAT CTC AC	
*OTUD1*	F: ATG GGG CAG ATG CTG AAT GTG A	G6	*USP28*	F: GAG GCA GCC CCA ACT GAA TC	G11
	R: TGC ACC AGT TGT CGT ACT CTG			R: TGC TCA GAT GAC AAG CAG CG	
*OTUD3*	F: GAA GAC GAC CTG AGA GAT GAA G	G7	*USP29*	F: GGG ATG ACT AAG CTG AAA GAA GCT	G10
	R: CTG GGC TCA AGA TTC TCT TCT G			R: TTT CAA AGT TAA ACG CAG GTG ACT	
*OTUD4*	F: GCT CTG CTA TGT GTC AGT CTC T	G7	*USP31*	F: TGA GGA TTG GTG TGG CCG TA	G11
	R: TTA CTT GCA ACT GTC ATC CTC TG			R: AAT CTT GTC GCT GCC TGC TC	
*OTUD5*	F: ATC GGA GGA GTC ATG GAT TGA A	G6	*USP33*	F: CCC TTG GTA CTT GTCA GGA TTG TA	G3
	R: ACC TGG CGA GCC TGT TTC TCC T			R: AAG CAT AAC ACC ATA CTC GAA GAG	
*OTUD6A*	F: TGG ATG ATC CGA AGA GTG AAC	G10	*USP34*	F: CAG CCA TAG TGC TGA AGT TCA AGT	G3
	R: TCT TGG AAC TTC TCC AGC TCC T			R: GAC TGA CAT CAC CAG ATT GTG CT	
*OTUD6B*	F: AAG AAT GCT GTT CCC AAG AAT G	G7	*USP35*	F: AAG TAC ATG CTC CTG ACC TTC CA	G8
	R: CCA TAT GTC TGG CTC CTG TTA A			R: CCC AGG TTG ATG AGA CCA ATC TT	
*OTUD7A*	F: GCA GCA CTT CTA CAT GAT CCT A	G8	*USP36*	F: TCC CAG ACA CCC ACA CAC AT	G12
	R: TGT GTA GAT TGG CAT CTC CAG G			R: GTG GTG TTG TCC GTG TCT G	
*OTUD7B*	F: ACT TCA CAG GGG TGC CTT GTT	G7	*USP37*	F: CAG AAG GAA ACC AGC AGG CA	G12
	R: GTT CTT CCC TGT AAC AAC AGG A			R: CGT CCG AGC TAT TCC ACT TCC	
*PARP11*	F: CAG CTA CAA GAT AGA CTT TGC AG	G7	*USP38*	F: CGT GTT GGG CCT CCT TCA TC	G11
	R: GAT GGC CTC GTT TTC ACA GAT G			R: TGC AGG GAA GGC AGT AGT GT	
*PRPF8*	F: TCT ATG ACG ACT GGC TCA AGA C	G5	*USP39*	F: GGA GTC TCG CGG TTC CAC T	G12
	R: ATC GCC ATG CTT GTT GAC AGT G			R: CGC ACA AAC GGG ACA ACA GA	
*PSMD14*	F: GGT TTG ACA CTT CAG GAC TAC A	G5	*USP41*	F: GGT TCT GCT TCA ATG ACT CCA ATA	G10
	R: GAG GTC ATA AGT ACA TCC ACAT G			R: AGC CAT CTC ACG ATT GAC CGG CT	
*PSMD7*	F: ACG TCT TCA ACC TGC TGC CAG A	G5	*USP42*	F: TTA CTC ATC CCA CCC ATA GCC	G1
	R: TCC TGC CCT TCT TTC TTC TCT G			R: TCA TGT GAG AGG GAA GCT GTG GT	
*STAMBP*	F: GAA GCC CTC CTT AGA TGT GTT	G4	*USP43*	F: GAC AGA GCT GTT TCC TGG GC	G11
	R: TGT CCA CCA CAG GTG GCT TAG CT			R: ATA GCT GCA GGC CAC AGA GA	
*STAMBPL1*	F: TTC GAA GAT CAA CTC AAG AAG CA	G5	*USP45*	F: TGG GCT GTT CAG ATC CAG TAG T	G1
	R: TCT GGT GTG TGG AAA AGC AGG A			R: ACT GTC AGT CTC CTT GGT GTA CAG	
*TNFAIP3*	F: CCG AGC TGT TCC ACT TGT TAA CA	G6	*USP46*	F: CCA ATC CTG CTG ATG TGG CAG TC	G2
	R: CAA CTT TGC GGC ATT GAT GAG A			R: GCT GAT GGC TGG AAA GAT GTA GTA	
*USP1*	F: GAC CAA ATG TGT GAA ATA GGT AAG C	G2	*USP47*	F: CAA TGA TCA ACA TGT CAG CAG GA	G1
	R: GCA AGT AAG GAG TAG AAG TAG GAG			R: TTT CTG GCT GGA TCC TTC AGT CT	
*USP2*	F: TAT GGT GCC TAC ACC CCG TCC T	G9	*USP48*	F: GCT GGT AGA TCG GGA TAA TTC CA	G2
	R: TGA GGA AGC TGC TGG TGG GGA C			R: AAC TCA TAG GGC TCA GCT CCA G	
*USP3*	F: CCT TGG GTC TGT TTG ACT TGT TCA	G3	*USP49*	F: AGG ACT ACG TGC TCA ATG ATA ACC	G9
	R: CCA GTC CCA GCT TGG TGT CAT TA			R: GCA GGA GCA GCC GTG CAC TCT	
*USP4*	F: GTA GAA GGC CAG CAA CCC ATC G	G1	*USP50*	F: CTA TGA TAC CCT TCC AGT TAA GG	G8
	R: ACT AGC ACC TGA CCC TGG TAT AG			R: TGG CAT TCA CGC AGC ATG TGT TG	
*USP5*	F: GTC CAC AAA GAC GAG TGC GCC T	G1	*USP51*	F: GGA CCC CAG AGA CTA GGA AAC G	G1
	R: AGG CTG AGT CGG CCG ACA GTA			R: CAT AAT CCT TAC ACA TGA AGC A	
*USP6*	F: CGT TGG AAT CAA CAG CAG CAT TGA	G10	*USP52*	F: TCT GGC AAG GTT TCC CTG AGA GA	G2
	R: CCA TCC ACT TGC TCG TTC GTG TCA			R: GGT TGC CAT GCA CAT CAA AGT CT	
*USP7*	F: CTC TCA GAC CAT GGG ATT TCC AC	G9	*USP53*	F: GAC ATT TCC AGA GAA TGT GCT CTG	G3
	R: ATT GGT GTG TAG ATA TGC CCA CAG			R: GAT CCA GAT TGG AAA TGT GAA AGG	
*USP8*	F: GAC GCC ACC TGC ATC TAT AGA AG	G1	*USP54*	F: CGT GGT AGT GTA CAA GGG ATG TTT	G2
	R: GGA AAG TAA AAC TGT CCT GCG CAA			R: CTC CCA TGC ACT TGT GAG TTG TAA	
*USP9X*	F: AGC TTC AAG GGT TCC AGG ACA AG	G1	*USPL1*	F: TCC CAA GTG ACA GAT AAA GAA GCT G	G12
	R: GAA GAC TAT CTC GCA ACA CTA TGG			R: ACC CAC AGA ACA CGA TGT TAA AGA	
*USP9Y*	F: GAG GCT GTG AGT GGC TGG AAG T	G9	*VCPIP1*	F: GCT CGC TAT GGA ATG GAC AAA C	G6
	R: CGG ACG TGT ACC ATT GTAAGA TAT G			R: ACA TGC TCT GGT TCT ATG AGG	
*USP10*	F: CCT CCA CAG CCC GCA GTA TAT TT	G3	*YOD1*	F: ACT TGC CCA TCC AAT CTG GTG A	G7
	R: GAG ATA GGA TCA TCG CCA CCA TCT			R: ACG TAA CTA GAA GCA CCA CGT T	
*USP11*	F: TGG TGG AAG GCG AGG ATT ATG TG	G2	*ZRANB1*	F: CTA GTG CAA GAC CAA GGG TG	G6
	R: GCT GGG CCA AGT GCC ATC TTT C			R: ACA CAT CTT TTA GCC TTG GCC C	

**Table 3:** RT-PCR and qRT-PCR primer set.

*DUB* Gene	Primer Sequence
*ATXN3L*	F: 5′-TCA GAA GAA AGT GAT GAG TCT GG-3′
	R: 5′-CTC TCA ATT GCT CTC GAA CTT G-3′
*USP12*	F: 5′-GAA CTC TGA GTC TGG TTA CAT CCT-3′
	R: 5′-GAG GAG CTG GTA TCT CTG ATT TCA-3′
*USP49*	F: 5′-CCC TGA ACG CTA TCA CTG CA-3′
	R: 5′-TTG GCC AGC ATC TCA GTG AG-3′
*COPS5*	F: 5′-GCA GTG GTG ATT GAT CCA ACA A-3′
	R: 5′-AGA CCT GAC CAG TGG TAT AGT C-3′
*USP34*	F: 5′-CAG CCA TAG TGC TGA AGT TCA AGT-3′
	R: 5′-GAC TGA CAT CAC CAG ATT GTG CT-3′
*GAPDH*	F: 5′-CCC TGA ACG CTA TCA CTG CA-3′
	R: 5′-CCA TCA CGC CAC AGT TTC-3′

## Supplementary Materials

Supplementary materials can be found at https://www.mdpi.com/1422-0067/21/5/1755/s1. Figure S1. Raw data of repeated multiplex RT-PCR used in this study with A2780 cells, Figure S2. Raw data of repeated multiplex RT-PCR used in this study with OVCAR5 cells, Figure S3. Raw data of repeated RT-PCR used in this study with ovarian cells, Figure S4. Raw data of repeated Western blot used in this study with ovarian cells, Table S1. qRT-PCR data used in this study.
