# Supplementary material for: Differential Expression of DUB Genes in Ovarian Cells Treated with Di-2-Ethylhexyl Phthalate
Source: Int J Mol Sci. 2020 Mar 4;21(5):1755. doi: 10.3390/ijms21051755 (PMC7084536; doi:10.3390/ijms21051755)
Supplement: Supplementary file 1 [file ijms-21-01755-s001.pdf]

Supplementary Information

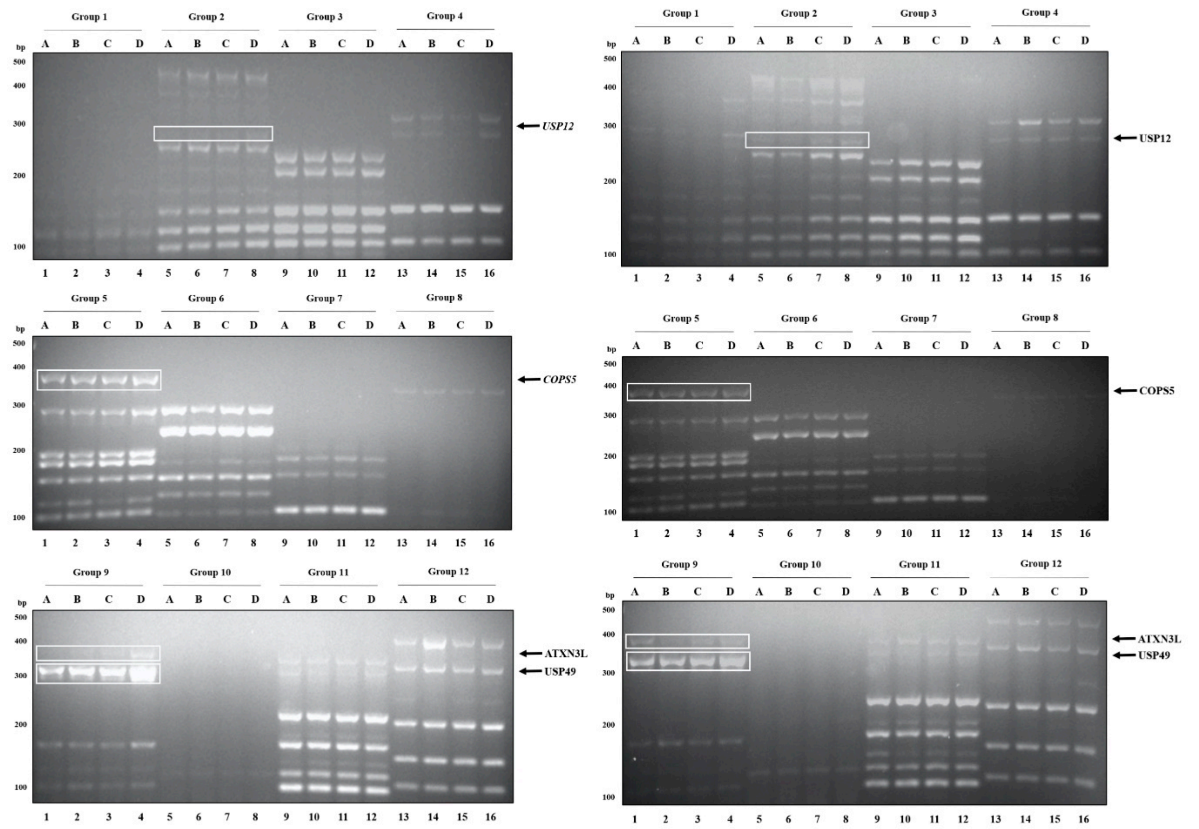

Figure S1. Raw data of repeated multiplex RT-PCR used in this study with A2780 cells.

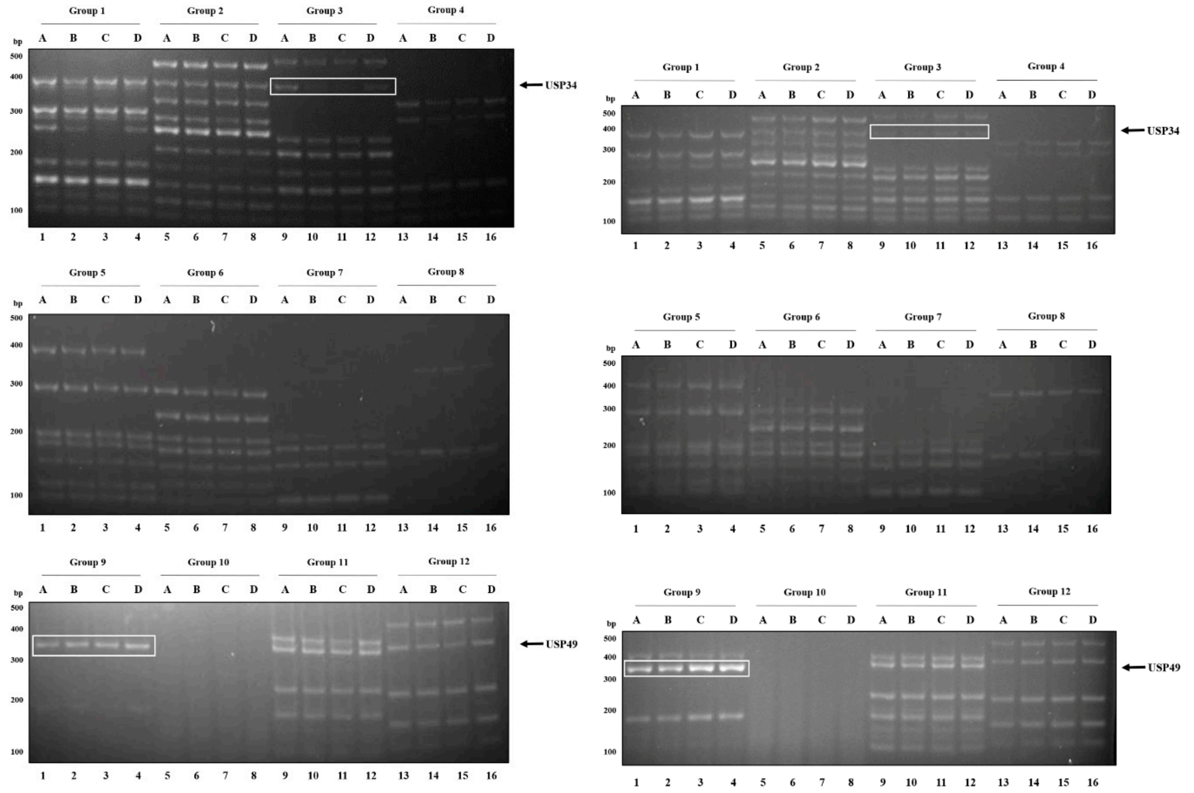

Figure S2. Raw data of repeated multiplex RT-PCR used in this study with OVCAR5 cells.

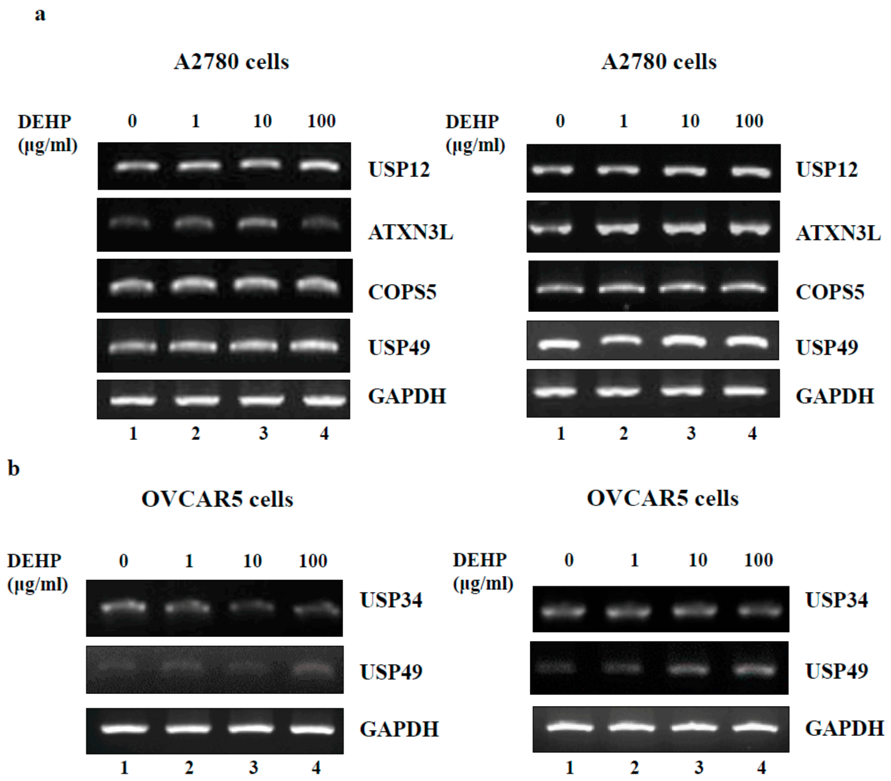

Figure S3. Raw data of repeated RT-PCR used in this study with ovarian cells.

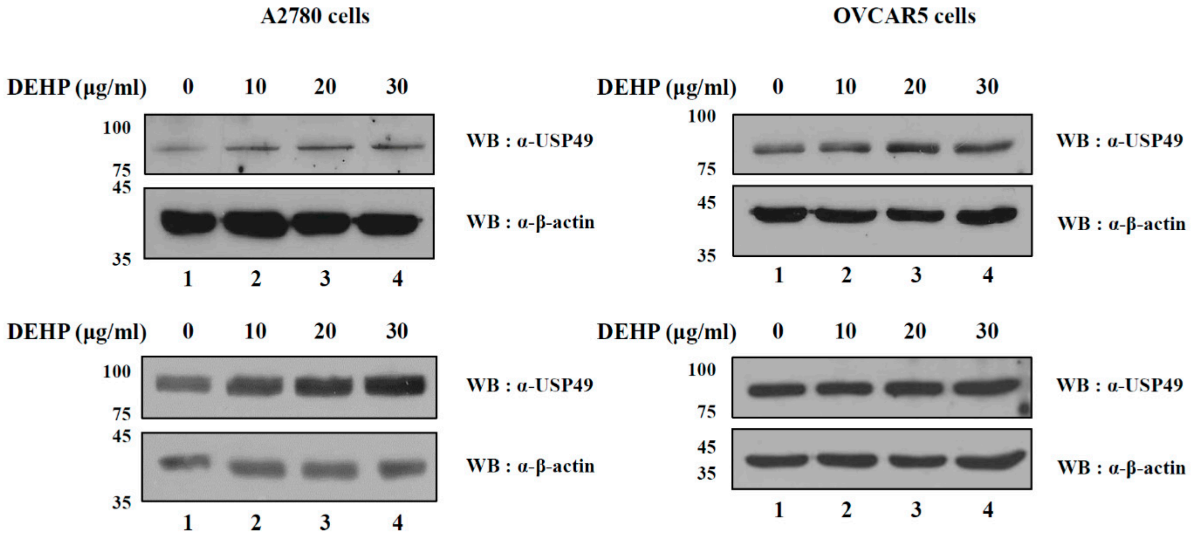

Figure S4. Raw data of repeated Western blot used in this study with ovarian cells.

Table S1. qRT-PCR data used in this study

Expression of *DUB* genes was normalized relative to normal condition (DEHP 0 μg/ml) $2^{-\Delta\Delta CT}$

cell line	gene	CT				ΔCT				ΔΔCT				2^-ΔΔCT			
		DEHP concentration (μg/ml)				DEHP concentration (μg/ml)				DEHP concentration (μg/ml)				DEHP concentration (μg/ml)			
		0	1	10	100	0	1	10	100	0	1	10	100	0	1	10	100
A2780	*USP12*	28.1206	28.3219	27.9947	26.714	10.4889	10.5511	10.2458	9.27275	0	0.0622	-0.2431	-1.2162	1	0.957801	1.183539	2.323309
		27.4484	27.056	26.9021	25.7387	9.81676	9.28529	9.15324	8.29746	0	-0.5315	-0.6635	-1.5193	1	1.445395	1.58394	2.866505
		27.6127	27.6017	27.1386	26.0671	9.98105	9.83097	9.38975	8.62587	0	-0.1501	-0.5913	-1.3552	1	1.109625	1.506601	2.558288
		27.4236	27.1051	26.7501	25.8842	9.79195	9.3344	9.00125	8.44296	0	-0.4576	-0.7907	-1.349	1	1.373208	1.729915	2.547341
	GAPDH mean	17.6317	17.7707	17.7489	17.4412												
	COPS5	24.183	23.8595	23.2072	23.12	5.76621	4.98858	4.78575	4.36075	0	-0.7776	-0.9805	-1.4055	1	1.714305	1.973088	2.649026
		24.1306	24.8474	24.0035	23.9181	5.71381	5.97643	5.58202	5.15888	0	0.26262	-0.1318	0.44507	1	0.833572	1.095654	0.734548
		24.0864	24.6215	23.6011	24.225	5.66953	5.7506	5.17965	5.46579	0	0.08106	-0.4899	-0.2037	1	0.945363	1.404335	1.151685
		24.1805	24.8848	23.9296	24.5078	5.76364	6.01387	5.5081	5.74854	0	0.25023	-0.2555	-0.0151	1	0.840764	1.193783	1.010522
	GAPDH mean	18.4168	18.8709	18.4215	18.7592												
	ATXN3L	33.2721	33.1073	33.4272	32.3807	15.6404	15.3365	15.6783	14.9395	0	-0.3039	0.03784	-0.7009	1	1.234484	0.974112	1.625519
		33.7672	33.6024	33.9223	32.8759	16.1356	15.8317	16.1734	15.4347	0	-0.5315	0.77611	-1.5193	1	1.445395	0.58394	2.866505
		33.5054	33.3332	33.9901	32.3164	15.8738	15.5624	16.2412	14.8752	0	-0.3114	0.36745	-0.9986	1	1.240874	0.775152	1.998025
	GAPDH mean	17.6317	17.7707	17.7489	17.4412												
	USP49	25.7181	26.321	25.6167	25.7919	8.10053	8.37154	8.0902	8.23932	0	0.271	-0.0103	0.13879	1	0.828743	1.007186	0.908283
		25.4583	25.5603	24.9752	24.6971	7.84075	7.61083	7.44866	7.14449	0	-0.2299	-0.3921	-0.6963	1	1.172766	1.312286	1.620297
		25.5211	25.8583	25.0076	24.8799	7.90353	7.90887	7.48109	7.3273	0	0.00533	-0.4224	-0.5762	1	0.996311	1.340195	1.490954
	GAPDH mean	17.6176	17.9494	17.5265	17.5526												
OVCAR5	USP34	24.1417	23.8217	25.2336	24.7856	6.63137	6.50547	7.51044	7.30508	0	-0.1259	0.87907	0.67372	1	1.091186	0.543716	0.62689
		24.2038	24.174	24.3199	24.2409	6.69348	6.85782	6.5967	6.76039	0	0.16435	-0.0968	0.06691	1	0.892332	1.069379	0.954679
		24.2913	24.0294	24.7013	24.4753	6.78103	6.71316	6.97808	6.99487	0	-0.0679	0.19705	0.21384	1	1.048168	0.872332	0.862238
	GAPDH mean	17.5103	17.3162	17.7232	17.4805												
	USP49	27.1287	26.9785	27.5719	27.3923	9.01342	8.94502	9.18682	9.09794	0	-0.0684	0.1734	0.08452	1	1.048554	0.886748	0.943098
		27.4329	26.7671	27.2376	27.5096	9.31756	8.73362	8.85247	9.21525	0	-0.5839	-0.4651	-0.1023	1	1.498936	1.380403	1.073493
		27.2456	27.3961	27.6019	27.3984	9.13026	9.36263	9.21686	9.10408	0	0.23237	0.0866	-0.0262	1	0.851237	0.941741	1.018315
		27.2808	27.1898	27.0152	27.2036	9.16549	9.15627	8.63008	8.90928	0	-0.0092	-0.5354	-0.2562	1	1.006414	1.449356	1.194336
	GAPDH mean	18.1153	18.0335	18.3851	18.2943												
